# Impaired antioxidant KEAP1-NRF2 system in amyotrophic lateral sclerosis: NRF2 activation as a potential therapeutic strategy

**DOI:** 10.1186/s13024-021-00479-8

**Published:** 2021-10-18

**Authors:** Silvia Bono, Marco Feligioni, Massimo Corbo

**Affiliations:** 1 Need Institute, Laboratory of Neurobiology for Translational Medicine, c/o Casa di Cura del Policlinico (CCP), Via Dezza 48, 20144 Milan, Italy; 2 Laboratory of Neuronal Cell Signaling, EBRI Rita Levi-Montalcini Foundation, 00161 Rome, Italy; 3 Department of Neurorehabilitation Sciences, Casa di Cura del Policlinico (CCP), Via Dezza 48, 20144 Milan, Italy

**Keywords:** Amyotrophic lateral sclerosis, Oxidative stress, KEAP1-NRF2, Neuroprotection, Therapeutic target, Antioxidant

## Abstract

**Background:**

Oxidative stress (OS) is an imbalance between oxidant and antioxidant species and, together with other numerous pathological mechanisms, leads to the degeneration and death of motor neurons (MNs) in amyotrophic lateral sclerosis (ALS).

**Main body:**

Two of the main players in the molecular and cellular response to OS are NRF2, the transcription nuclear factor erythroid 2-related factor 2, and its principal negative regulator, KEAP1, Kelch-like ECH (erythroid cell-derived protein with CNC homology)-associated protein 1. Here we first provide an overview of the structural organization, regulation, and critical role of the KEAP1-NRF2 system in counteracting OS, with a focus on its alteration in ALS. We then examine several compounds capable of promoting NRF2 activity thereby inducing cytoprotective effects, and which are currently in different stages of clinical development for many pathologies, including neurodegenerative diseases.

**Conclusions:**

Although challenges associated with some of these compounds remain, important advances have been made in the development of safer and more effective drugs that could actually represent a breakthrough for fatal degenerative diseases such as ALS.

## Background

Oxidative stress (OS) is a toxic cellular status due to the imbalance between oxidant and antioxidant mechanisms resulting from increased production of oxidants, a dysfunction in the antioxidant system, or both [1, 2]. OS can be triggered by free radicals or other oxidant species produced by either exogenous processes (environmental pollutants, drugs, viruses, bacteria, ionizing radiations, heavy metals, poor diet, and smoking) [3–11] or endogenous processes (such as immune cell activation, inflammation, ischemia, infection, cancer, and aging) [12–19].

**Fig. 1:**
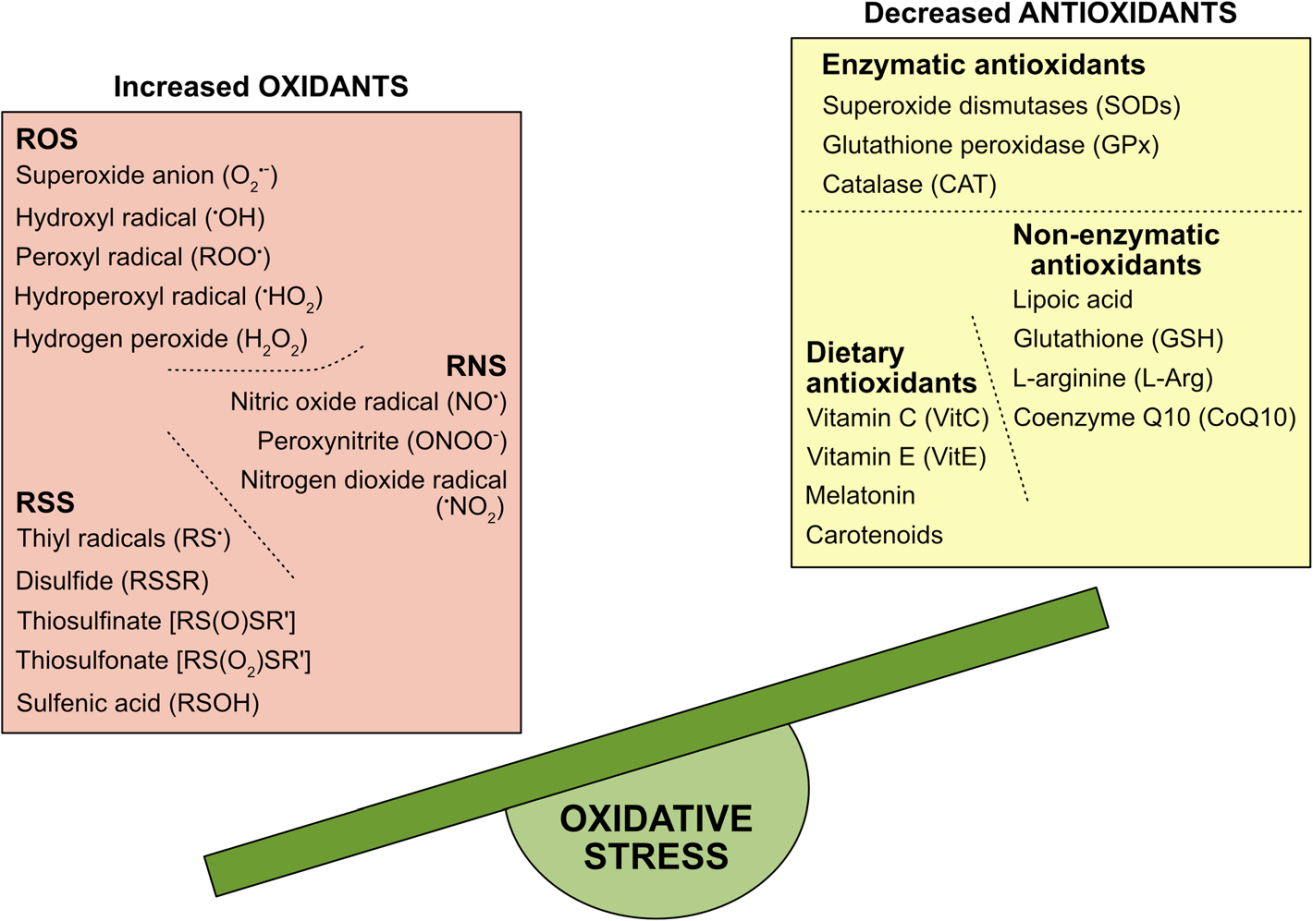
Redox imbalance in OS. An increased production of oxidants (ROS, RNS, and RSS) and a dysfunction, mostly a decrease, in the antioxidant system (Enzymatic, Non-enzymatic, and Dietary antioxidants) result in damage to cells and can contribute to the pathogenesis and progression of many neurodegenerative diseases, including ALS. ROS: reactive oxygen species, RNS: reactive nitrogen species, RSS: reactive sulfur species. ^•^Refers to an unpaired electron; R and R' represent functional groups ≠ H

Free radicals are molecules that are highly reactive to other cellular structures due to the presence of unpaired electrons [16, 20–22]. Oxygen (O_2_) is essential for life and plays a crucial role in many biological processes [23] and, given to its high oxidation-reduction (redox) potential, it is an ideal electron acceptor. However, its reactivity has a cost: O_2_ strips electrons from biological macromolecules causing the formation of reactive oxygen species (ROS), which induce intracellular damage [24]. Among them, the best known ROS in human cells include superoxide anion (O_2_^•-^), which is the precursor of most other ROS, hydroxyl radical (^•^OH), peroxyl radical (ROO^•^), hydroperoxyl radical (^•^HO_2_), and hydrogen peroxide (H_2_O_2_), although the last one is not technically a free radical because it does not have unpaired electrons, but it represents a source of them [25] (Fig. 1). Several sub-cellular compartments generating ROS have been identified, including cytosol, peroxisomes, plasma, and endoplasmic reticulum (ER) membranes [26, 27]. Nevertheless, under physiological conditions, in most tissues mitochondria are the major contributors to the cellular ROS production [28–31], mainly as a side effect of aerobic respiration via oxidative phosphorylation (OXPHOS) occurring on the inner membrane of the mitochondrion [32, 33], but also through other metabolic routes, such as pentose-phosphate pathway and glutathione (GSH) metabolism [34].

Although oxidative damage in biological systems has in the past been completely attributed to ROS, it is becoming increasingly clear that also reactive nitrogen species (RNS) and reactive sulfur species (RSS) are integral components of signal transduction processes in cells, and play important roles in OS generation [2, 25, 35]. The predominant RNS is nitric oxide radical (NO^•^) [36], formed in biological tissues from the oxidation of L-arginine to citrulline by nitric oxide synthase [37, 38], and precursor of other two RNS, the peroxynitrite (ONOO^−^), and the nitrogen dioxide radical (^•^NO_2_) (Fig. 1). The latter was once believed to predominantly be an environmental pollutant [39] but, it has been extensively demonstrated to be also produced endogenously by a subtype of white blood cells [40, 41]. The main sulfur-centered reactive species, RSS, are the products of oxidation of the thiol groups in proteins [2, 35, 42] (Fig. 1). RNS and RSS both contribute to fluctuations in redox homeostasis within the cells and, like ROS, are mainly produced within mitochondria [42–45]. Basal levels of ROS, RNS, and RSS are physiologically generated in cells and serve important regulatory and mediator functions. However, an uncontrolled increase in their concentrations leads to a chain of unwanted radical reactions that target the main biomolecules (DNA, RNA, lipids, and proteins) and alters key processes such as nucleic acid oxidation, lipid peroxidation, thereby contributing to the loss of cellular architecture, to impaired cell signalling and, ultimately, to cell death [25, 46].

Evidence suggests that OS is a common signature in neurodegenerative diseases such as Alzheimer’s disease (AD) [47–49], Huntington’s disease (HD) [50–52], Parkinson’s disease (PD) [53–55] and, in particular, it is part of the tightly connected and multi-factorial set of mechanisms involved in the pathogenesis and progression of Amyotrophic lateral sclerosis (ALS) [25, 56–58]. For counteracting OS effects, cells activate multiple antioxidant defense systems based on both enzymatic and non-enzymatic components. Enzymatic activities include superoxide dismutase (SOD), glutathione peroxidase (GPx), catalase (CAT); some of the important non-enzymatic antioxidant molecules endogenously produced are lipoic acid, reduced GSH, L-arginine (L-Arg), and coenzyme Q10 [59–62]. Together with dietary antioxidants such as Vitamin C, Vitamin E, melatonin, and carotenoids [63, 64], these antioxidant systems serve as “free radical scavengers” by preventing, buffering, and repairing damages caused by OS, and enhancing the immune defense against degenerative diseases [65] (Fig. 1).

In that regard, the transcription factor NRF2 (nuclear factor erythroid 2-related factor 2) and its main negative regulator KEAP1 (Kelch-like ECH (erythroid cell-derived protein with CNC homology)-associated protein 1) represent a finely regulated antioxidant system, with a key role in the response to OS and maintenance of redox homeostasis. KEAP1 acts as an OS sensor to regulate NRF2 turnover within the cell/body, while NRF2 activation leads to the production of antioxidant and detoxifying substances that decrease OS [66–68].

This review aims to highlight the central role of OS in the pathophysiology of ALS, specifically focusing on the KEAP1-NRF2 system and its alteration in ALS, while discussing the potential applications of NRF2 activators in ALS therapy.

## Main text

### Role of oxidative stress in ALS

ALS is an adult-onset neurodegenerative disease, characterized by degeneration and loss of upper motor neurons (MNs) in the cerebral cortex and lower MNs in the brainstem and spinal cord [69], leading to progressive weakness of voluntary muscles, and death caused by a diaphragmatic failure within 2–5 years [70, 71]. Most of the cases of ALS are sporadic (sALS) with no genetic linkage, while around 10% are associated with familial forms (fALS), presenting mutations in over 20 genes. The most common of them include Cu^2+^/Zn^2+^ *superoxide dismutase 1* (*SOD1*), *TAR DNA-binding protein 43* (*TARDBP)*, *fused in Sarcom*a (*FUS*)*,* and *chromosome 9 open reading frame 72* (*C9orf72)* genes, involved in protein homeostasis and RNA metabolism [72–75]. sALS and fALS’ progression is clinically and pathologically very similar, both characterized by the progressive accumulation of toxic misfolded protein aggregates within cells [76] and the significant loss of MNs [77]. To date, there is still no effective treatment for ALS, and the two currently available drugs, riluzole and edaravone, only extend survival of ALS patients by few months [78–83].

The precise regulation of ALS pathogenetic mechanisms is still elusive, despite the abundance of identified mechanisms mediating MN degeneration and death, including neuroinflammation, glutamate excitotoxicity, altered RNA metabolism, mitochondrial dysfunction, impaired cytoskeletal integrity, altered axonal transport dynamics, OS [71, 84, 85].

Given the complexity of the disease, it is therefore not surprising that dissecting cause and effect is not a simple matter. The role of OS in ALS pathology, whether as a primary cause of disease or a secondary consequence, is now well recognized and many preclinical and clinical studies have consistently demonstrated that the disease is characterized by high levels of OS markers able to induce cellular damage, impaired cell signalling, and finally cell death [25, 86]. So, it is likely that OS can be triggered by following an increase in the production of O2^•-^ and NO^•^ in MNs as well as in the central glia, starting from the presymptomatic phase of ALS. This unfavorable condition could also be facilitated by the early reduction of the GSH level in the various tissues of subjects affected by ALS [87]. However, as the main evidence comes from post mortem tissues, the temporal evolution of OS markers in ALS is not yet well defined.

Early evidence demonstrated that high levels of protein carbonyl groups, a general marker of OS, were present in both postmortem spinal cord [88, 89] and motor cortex [90] from sALS patients, compared to control samples. Likewise, increased 3-nitrotyrosine (3-NT) levels, a marker for peroxynitrite-mediated damage, were observed within spinal cord MNs [91]. High levels of lipid peroxidation-derived aldehydes, including 4-hydroxy-nonenal (4-HNE) and malondialdehyde (MDA) [92], protein glycoxidation markers such as pentosidine [93], and stress-induced DNA damage molecules, more specifically 8-hydroxy-2′-deoxyguanosine (8-OHdG) [94, 95], have also been revealed in the spinal cord from sALS patients. In addition, high levels of most of these OS biomarkers [95–98], together with high levels of ascorbate free radical [96], and two products of NO oxidation, such as nitrite and nitrate [99], were also detected in cerebrospinal fluid (CSF) samples from ALS patients, showing a positive correlation with 8-OHdG level and disease severity over the 9-month duration of the prospective study [95]. These findings were consistent with the hypothesis that oxidative pathology accompanies the neurodegenerative process in ALS and suggest that 8-OHdG could be a sensitive biomarker to be dynamically quantified during ALS to gather more information on the temporal evolution of OS and on disease progression.

While OS is recognized as an important feature in ALS, the role of impaired GSH metabolism is still debated. In two studies no detectable [100] or unchanged [101] activities of GSH peroxidase were found in spinal cord or cerebral cortex of ALS patients. Some other studies, on the contrary, have revealed a decrease in the content of antioxidant GSH. In particular, back in 1996 Przedborski and colleagues indicated that GSH peroxidase activity is reduced in a specific brain region affected in ALS, the precentral gyrus, suggesting that the observed deficit could be involved in the oxidative damage observed in sporadic ALS [102]. In another study they described a similar GSH reduction in red blood cells of sALS patients treated with insulin-like growth factor 1 (IGF-1), but probably only as a secondary cytotoxic effect due to the treatment with IGF-1 [103]. The oxidant-antioxidant imbalance in the erythrocytes of sALS patients was confirmed, more recently, in another study where GSH reductase and reduced GSH levels were also found to be significantly lower at 24 months from the onset of ALS, in patients who died shortly after [104]. In addition, a study performed along 72 months has shown a significant impairment of erythrocytes GSH peroxidase activity especially in subjects with a faster rate of disease progression [105]. Similarly, the first in vivo imaging studies in humans, confirmed an increase in OS and a simultaneous decrease in GSH levels in the motor cortex of patients with ALS (with an age of 65.2 ± 9.4 years, and a mean disease duration of 25.8 ± 17.1 months) compared to the controls [106, 107]. Furthermore, the fact that tracer retention in cortical regions increased with clinical disease severity, as estimated by the Revised ALS Functioning Rating Scale (ALSFRS-R), suggested that OS may be an important factor associated with the development of neurodegeneration in ALS patients [107]. Then in 2019, a meta-analysis study tried to clarify the OS marker profile in ALS patients: the authors demonstrated that 8-OHdG and MDA were significantly increased in the peripheral blood of ALS patients when compared with control subjects, while GSH levels were significantly reduced. However, due to the heterogeneity between studies and other clinical variables, more studies are needed to better understand how prooxidative imbalances contribute to the pathophysiology of ALS [108].

**Fig. 2:**
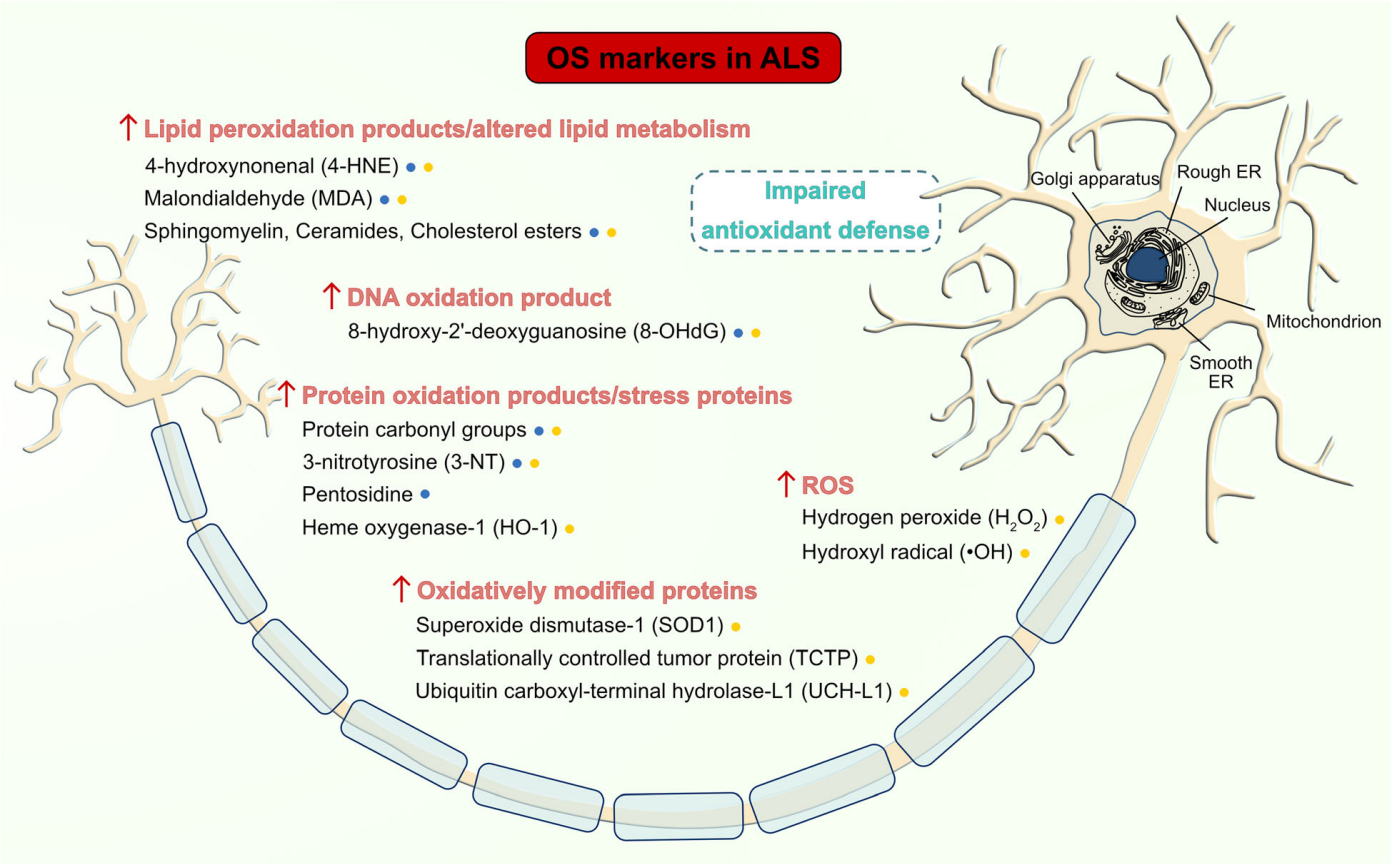
OS markers in ALS. Increased levels of products of lipid peroxidation (and altered lipid metabolism), DNA oxidation, protein oxidation, as well as stress proteins, oxidatively modified proteins, and ROS have been found in ALS patients (*blue points*) and in SOD1^G93A^ transgenic mice (*yellow points*); the presence of impaired antioxidant defense has been highlighted

In line with patient studies, cellular and mouse models of ALS also showed high levels of ROS, along with elevated oxidation of membrane phospholipids (HNE, MDA), DNA (8-OHdG), and proteins (protein carbonyl groups, 3-NT) [94, 109–111]. Another preclinical study using presymptomatic and symptomatic SOD1^G93A^ transgenic mice revealed an association between the accumulation of ceramides and cholesterol esters in the spinal cords of ALS patients and OS, thus highlighting the pivotal role of OS in inducing an alteration in the metabolism of sphingolipids metabolism which mediates MN death in ALS [112]. To further strengthen the contribution of OS in ALS, an increase in the level of several proteins oxidatively modified and consequently inactivated has been found in SOD1^G93A^ transgenic mice and correlated with SOD1 mutation: such proteins are SOD1 itself, translationally controlled tumor protein (TCTP, which normally processes calcium binding activity and acts as a cytoprotective factor), and ubiquitin carboxyl-terminal hydrolase isoenzyme L1 (UCH-L1, which plays an important role in the ubiquitin-proteasome system, UPS). Both oxidatively modified SOD1 and UCH-L1 are involved in the formation of cytoplasmic inclusions in ALS mouse models and in ALS patients [113]. Taken together, these findings indicate a potential relationship between protein oxidation, Ca^2+^ regulation, and protein aggregation in ALS (Fig. 2).

Overall, considering the evidence reported here, the cellular antioxidant defense in ALS appears to be strongly diminished, with an imbalance towards high and persistent OS levels. Therefore, a better understanding of the dysregulation of the antioxidant KEAP1-NRF2 activity in ALS could help to develop novel therapeutic strategies able to halt disease progression, prolonging patients’ survival.

### KEAP1-NRF2: the cellular response to OS

#### Structural insights

KEAP1 and NRF2 are two relevant players in the molecular and cellular response to OS [114, 115].

**Fig. 3:**
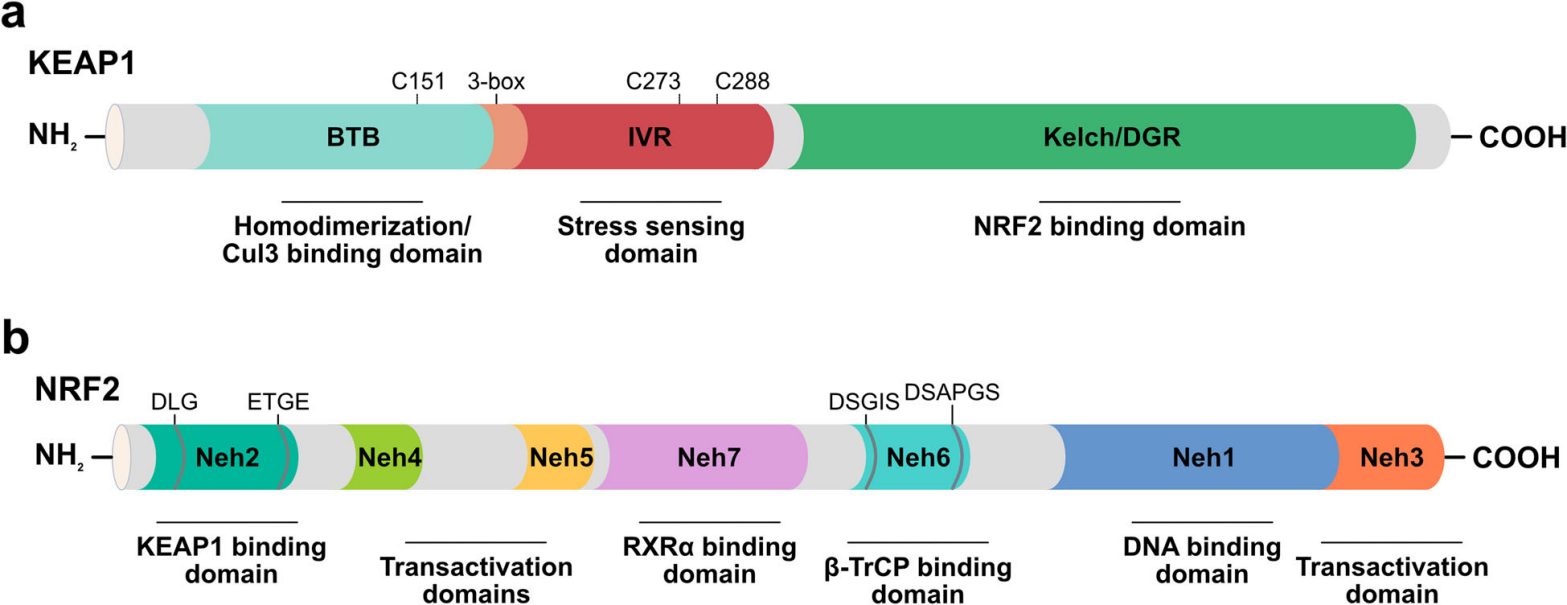
Domain architecture of the human KEAP1 (a) and NRF2 (b) proteins. Functional domains, relevant motifs (3-box, DLG, ETGE, DSGIS, DSAPGS), and the most important cysteine residues (C151, C273, C288) involved in stress sensing are indicated. BTB: Broad complex/Tramtrack/Bric à Brac, IVR: intervening region, DGR: double glycine region, Neh1–7: NRF2-ECH homology 1–7

KEAP1 belongs to the BTB-Kelch family of proteins, known to be involved in ubiquitination [116, 117]. It was cloned for the first time using the Neh2 domain of NRF2 as bait in a yeast two-hybrid system by Yamamoto and coworkers [118]. It acts as a substrate linker for the interaction of Cullin 3 (CUL3)-dependent E3 ubiquitin ligase complex [119], with NRF2 leading to continuous ubiquitination and proteasomal degradation of NRF2 [120], thus representing the main negative regulator of NRF2 activity. Three major functional domains represent the essence of the KEAP1 protein structure: the N-terminal Broad complex/Tramtrack/Bric à brac (BTB) domain, the central intervening region (IVR) and the C-terminal Kelch/Double glycine repeat (DGR) domain [121, 122]. The BTB domain binds Cul3, contains the Cysteine151 residue (C151), which is one of the important cysteine residues in stress sensing, and mediates KEAP1 homodimerization [123–125]. The IVR domain contains a 3-box motif in its proximal part, which provides an additional CUL3 interaction site [116], and two critical cysteine residues, Cysteine273 (C273) and Cysteine288 (C288), that are important for the repression of NRF2 activity [126]; IVR connects the BTB domain with the Kelch/DGR domain. The latter domain is required for substrate capture and can bind separately to both DLG (Aspartic acid-Leucine-Glycine) and ETGE (Glutamic acid-Threonine-Glycine-Glutamic acid) motifs on the NRF2-ECH homology (Neh) 2 domain of NRF2 [127–129] (Fig. 3a).

NRF2 is a member of the cap ‘n’ collar (CNC) subfamily of the basic region leucine zipper (bZIP) transcription factors, involved in multiple cellular processes [130–135]. Among them, its most prominent role is to induce the antioxidant responsive element (ARE)-mediated genes in response to OS, thereby ensuring cytoprotection [136]. In fact, NRF2 deletion causes an increase in OS and cellular death both in vitro and in vivo [137, 138]. The *Nuclear factor erythroid 2 like 2* (*NFE2L2*) gene, encoding NRF2 protein, was cloned as a factor that binds to the *β-globin* promoter [139, 140]. Moving from the N-terminal to the C-terminal region, the protein structure includes seven highly conserved Neh domains [121, 122, 141, 142]. The Neh2 domain is a regulatory domain containing the DLG and ETGE binding motifs, through which NRF2 interacts with its major negative regulator, KEAP1, and is essential for the KEAP1-mediated degradation of the protein by the proteasome [128, 143–145]. The Neh4 and Neh5 domains are two important transactivation domains; they recruit cAMP response element-binding protein (CREB)-binding protein (CBP) or receptor-associated coactivator 3 (RAC3), to facilitate the transactivation of NRF2 target genes [146, 147]. The Neh7 domain mediates the binding to retinoid X receptor alpha (RXRα), another negative regulator of NRF2 [148]. The Neh6 domain, which is a serine-rich region, contributes to regulate NRF2 stability and mediates the interaction with a third negative regulator, β-transducin repeat-containing protein (β-TrCP), through its DSGIS (Aspartic acid-Serine-Glycine-Isoleucine-Serine) and DSAPGS (Aspartic acid-Serine-Alanine-Proline-Glycine-Serine) motifs not recognized by KEAP1. Phosphorylation of the DSGIS motif in NRF2 mediated by glycogen synthase kinase-3 beta (GSK-3β) promotes the binding of β-TrCP to NRF2 which is therefore marked for ubiquitination and then degraded by the proteasome [145, 149–151]. The Neh1 domain contains a bZIP binding motif responsible for the formation of a heterodimer with small musculoaponeurotic fibrosarcoma (sMaf) proteins and mediates the binding to ARE sequences, within the promoter of NRF2 target genes [152]. Finally, the C-terminal Neh3 domain is another transactivation domain that recruits chromo-ATPase/helicase DNA-binding protein 6 (CHD6), functioning as an NRF2 transcriptional coactivator [153] (Fig. 3b).

#### Spatiotemporal regulation of the KEAP1-NRF2 system

**Fig. 4:**
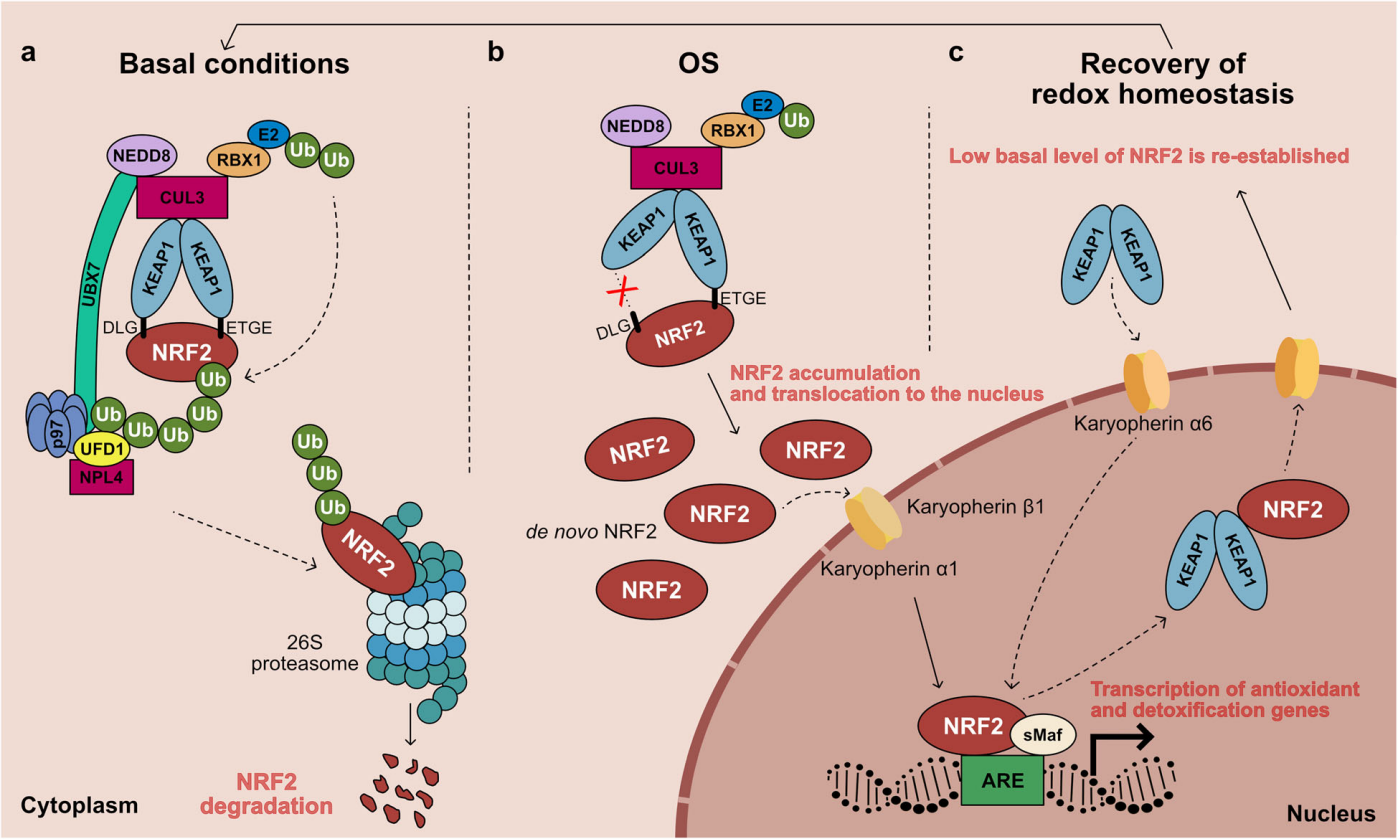
Schematic model of the KEAP1-NRF2 signalling pathway under basal conditions (a), OS (b), and after the recovery of cellular redox homeostasis (c). (a) Under basal conditions NRF2 is sequestered by the KEAP1-NEDD8-CUL3-RBX1 complex in the cytoplasm, transferring ubiquitin (Ub) proteins from E2 ligase to the Neh2 domain in NRF2. In addition, the UBX7-p97-UFD1/NPL4 complex interacts with ubiquitinated NRF2 and NEDD8-CUL3 complex and transfers NRF2 to 26S proteasome for its degradation. (b) OS causes the oxidation of cysteine residues in KEAP1, inducing a conformational change in its structure and preventing NRF2 ubiquitination. NRF2 is therefore stabilized and translocates to the nucleus, where it binds to sMaf proteins activating ARE-driven genes. (c) Upon the recovery of redox homeostasis, KEAP1 translocates into the nucleus and induces NRF2 nuclear export. In the cytosol, NRF2 is ubiquitinated and degraded, and its level returns to be physiologically low

Under physiological conditions, KEAP1 is the main regulator of the NRF2 protein level (NRF2 has a half-life of about 20 min) [120, 145]. More specifically, KEAP1 homodimerizes [124], binds to the ETGE and DLG motifs on Neh2 domain of NRF2, and also acts as a substrate adaptor protein [154] for the KEAP1-NEDD8-CUL3-RBX1 E3 ubiquitin ligase complex [119, 120, 155]. CUL3 is linked to the ubiquitin-like protein NEDD8 [156] and represents a scaffolding protein between KEAP1 and RBX1. This conformation of KEAP1-NRF2 complex allows an adequate Lysine (Lys) rich α-helix orientation in the Neh2 domain for KEAP1-dependent polyubiquitination [157]. In addition, the UBX7-p97-UFD1/NPL4 complex, consisting of a ubiquitin-targeted ATP-dependent segregase, p97, and two heterodimeric cofactors, UBX7 and UFD1/NPL4, extracts ubiquitinated NRF2 from the KEAP1-NRF2 complex and transfers it to the 26S proteasome for its degradation [154] (Fig. 4a).

In response to OS, different metabolites oxidize highly reactive cysteine residues in KEAP1, inducing a conformational change in the KEAP1-NEDD8-CUL3-RBX1-E3 ubiquitin ligase complex, with an improper orientation of Lys residues on NRF2 [122, 158]**.** This leads to impaired ubiquitination of NRF2, an increase of NRF2 protein levels [157], and a reduction of KEAP1 levels able to sequester de novo synthesized NRF2 [159]. NRF2 therefore accumulates and is free to translocate to the nucleus through the nuclear pore complex (NPC). This translocation is supported by Karyopherin α1 (importin α5), that functions as an adaptor molecule, and Karyopherin β1 (importin β1), the transport receptor that allows NRF2 translocation from the cytoplasm to the nuclear side of the NPC, due to Nuclear Localization Signal (NLS) sequences in NRF2 [160]. NRF2 then dimerizes with members of the sMaf proteins through its bZIP domains and binds to the ARE sequences, which are regulatory enhancers, within gene promoters [156, 161]. Classical NRF2 target genes encoding antioxidant and detoxifying enzymes are responsible for maintaining redox balance [162]. Some examples include: NADPH quinone dehydrogenase (NQO1), heme oxygenase 1 (HO1), the two subunits of glutamate-cysteine ligase (GCL), glutamate-cysteine ligase modifier (GCLM, the rate-limiting enzyme for GSH synthesis), SOD 1, CAT, sulfiredoxin, thioredoxin, and peroxiredoxin [68, 137, 162, 163]. In addition, NRF2 is capable of increasing the expression of many autophagy-related genes that present a common ARE sequence in their promoters, including *Sequestosome-1* (*SQSTM1*) gene (encoding protein 62) [164], and of attenuating inflammation by blocking pro-inflammatory cytokine (IL-6 and IL-1β) transcription [132], thus highlighting its crucial contribution to different cellular pathways. Furthermore, NRF2 controls genes encoding enzymes governing different metabolic pathways, such as lipogenesis and lipid degradation, lipid transport and uptake, enzymes of the pentose phosphate pathway, and some enzymes of glycogen metabolism, like glycogen branching enzyme (GBE) and phosphorylase b kinase subunit A1 (PHKA1), recently linked to NRF2 signalling in the muscle [165] (Fig. 4b). After the recovery of cellular redox homeostasis, KEAP1 translocates into the nucleus through Karyopherin α6 (importin α7), inducing NRF2 nuclear export to the cytosol, in a CRM1-dependent process through Nuclear Export Signal (NES) sequences present in NRF2. Low basal level of NRF2 is therefore re-established by KEAP1-mediated ubiquitination and degradation, and the NRF2 signalling pathway is turned off [166–168] (Fig. 4c).

### Regulatory mechanisms

**Fig. 5:**
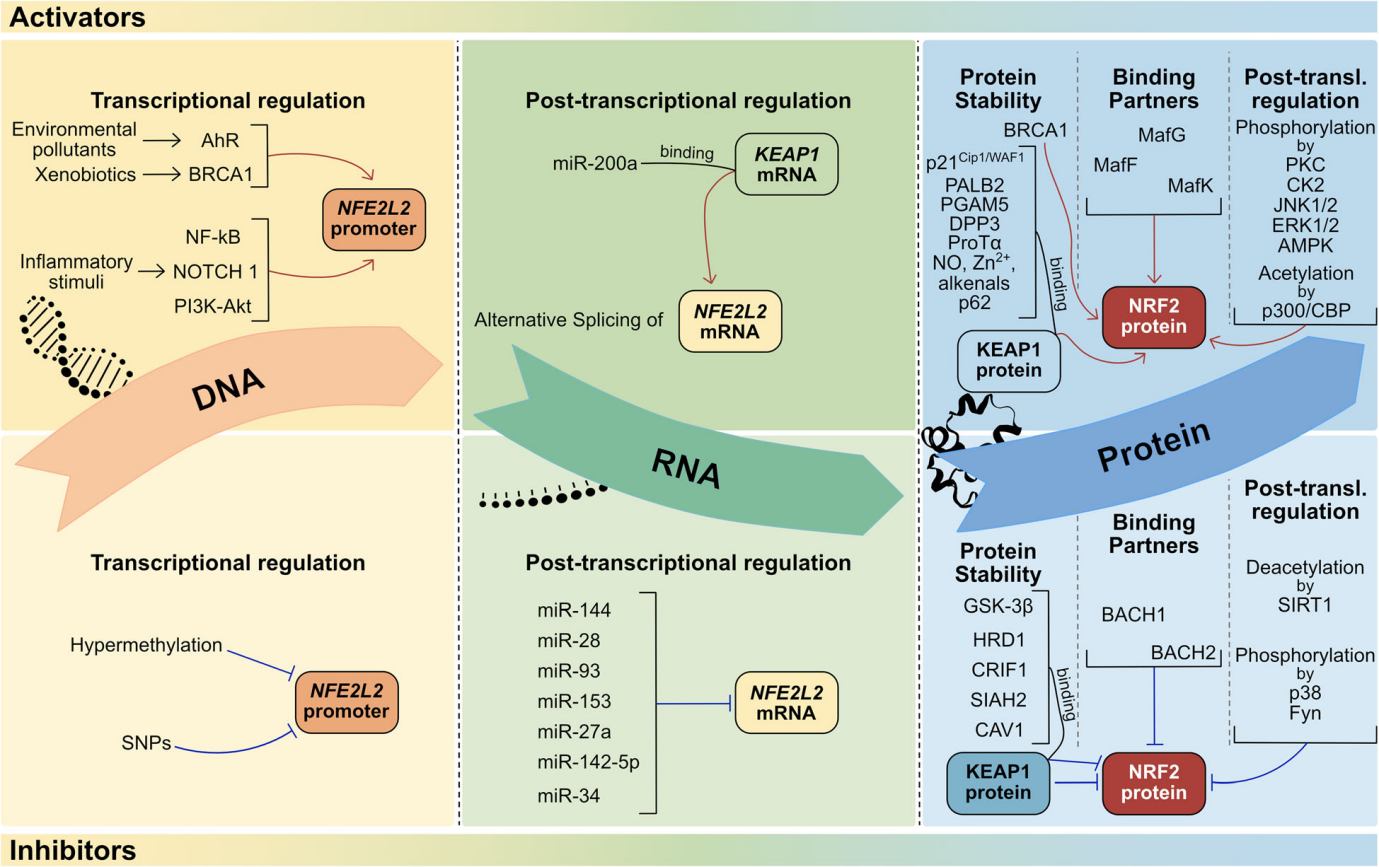
Schematic overview of the cellular mechanisms and processes modulating the KEAP1-NRF2 system. Activators (upper panels) and inhibitors (bottom panels) tightly modulate KEAP1-NRF2 activity at the level of DNA, RNA, and protein: transcriptional regulation, post-transcriptional regulation, protein stability, availability of binding partners, and post-translational regulation are involved. Red arrows indicate the activating effect, while blue bars indicate an inhibitory effect

Many cellular mechanisms and processes tightly modulate the activity of KEAP1-NRF2 system at multiple levels [169]. More specifically, a complex regulation at the DNA level (transcriptional control), at the mRNA level (post-transcriptional control), and at the protein level (protein stability, availability of binding partners, and post-translational control) is responsible for maintaining cellular redox homeostasis (Fig. 5). A better understanding of the detailed interactions between the factors involved will contribute to further elucidate the cell defense capability, which is impaired in neurodegenerative diseases such as ALS [67, 141, 170], and may reveal the path for identifying novel potential therapeutic targets.

#### Activators

At the transcriptional level, *NFE2L2* gene is induced by several transcription factors, including the aryl hydrocarbon receptor (AhR) in response to environmental pollutants like polycyclic aromatic hydrocarbons, breast cancer susceptibility gene 1 (BRCA1) in response to xenobiotic stress, and the nuclear factor kappa-light-chain-enhancer of activated B cells (NF-κB) in response to inflammatory stimuli. The first two bind to the xenobiotic response element (XRE) sequences, while the last binds to the NF-κB binding site in the *NFE2L2* promoter, inducing its transcription [171–174], with a consequent increase of *NFE2L2* mRNA available within the cell. To note, NRF2 regulates BRCA1 by the presence of an ARE sequence in *BRCA1* promoter, creating a positive-feedback loop, with an increase of both transcripts [175]. Additionally, the neurogenic locus notch homolog protein 1 (NOTCH1) signalling pathway [133] and the phosphoinositide 3-kinase (PI3K)-protein kinase B (AKT) pathway [176, 177], have also been shown to augment *NFE2L2* transcription, and consequently activate NRF2 signalling, thus maintaining redox balance.

At the post-transcriptional level, it has been demonstrated that the KEAP1-NRF2 system can be positively controlled by a microRNA (miRNA) able to target *KEAP1* gene [178]. miRNAs are small (20–23-nucleotides), endogenous, single-stranded noncoding RNAs that regulate gene expression by pairing to sequence-specific binding sites in mRNAs to direct post-transcriptional repression and consequent inhibition of protein translation or degradation of the mRNA targets [179–181]. In particular, Eades’group in 2011 identified and validated for the first time in MDA-MB-231 and Hs578T breast cancer cells a miRNA, miR-200a, able to target the *KEAP1* 3′-untranslated region (3′-UTR), leading to *KEAP1* mRNA degradation and, consequently, NRF2 stabilization and translocation to the nucleus [178]. MiR-7 also activates NRF2 by targeting KEAP1 expression in human SH-SY5Y neuroblastoma cells, resulting in increased levels of the reduced form of GSH, indicative of OS mitigation [182]. Another post-transcriptional mechanism that can regulate NRF2 is alternative splicing. In fact, it has been observed that aberrant *NFE2L2* transcript variants missing exon 2, or exons 2 and 3, are present in lung cancers and head and neck cancers. The NRF2 protein isoforms encoded by these splice variants lack the KEAP1 interaction domain, thus preventing NRF2 degradation and resulting in protein stabilization and transcription of NRF2 target genes [183].

Like many other stress responsive transcription factors, NRF2 is mainly regulated at the level of protein stability by a variety of proteins; some of them interact directly with NRF2, while others interact indirectly with it and directly with KEAP1 [169]. More precisely, among them, the cyclin-dependent kinase inhibitor p21^Cip1/WAF1^, induced by p53 in response to OS and involved in the regulation of cell cycle arrest, replication, cellular proliferation, and apoptosis, competes with KEAP1 for the binding to NRF2 through the DLG motif, thus preventing NRF2 ubiquitination, and increasing its stabilization [184, 185]. BRCA1, which regulates cell cycle progression, DNA damage signalling and repair, and maintenance of genomic integrity, interacts with NRF2 either directly, through its ETGE motif, preventing KEAP1-dependent ubiquitination of NRF2 and protecting cells against OS, or indirectly, through the interaction with CBP or with c-Myc [186]. Other proteins that bind KEAP1 and interfere with KEAP1-NRF2 interaction, promoting NRF2 accumulation and function in the nucleus, include: phosphoglycerate mutase 5 (PGAM5, a serine/threonine phosphatase related to the phosphoglycerate mutase family), which has been reported to be associated with mitochondria, so its interaction with KEAP1 may be regulated by mitochondrial signalling processes [187, 188]; partner and localizer of BRCA2 (PALB2), which is a nuclear protein playing an important role in maintaining genome integrity [189]; dipeptidyl-peptidase 3 (DPP3), which is a zinc aminopeptidase involved in protein turnover [190, 191]; prothymosin alpha (ProTα, a nuclear protein involved in cellular proliferation and protection against apoptosis [192], whose interaction with KEAP1 has been found increased after cell treatment with the OS inducer diethyl maleate (DEM) [193, 194]. NRF2 activity is also increased by stress associated chemicals, such as NO, Zn^2+^, and alkenals (a class of carbonyls produced by lipid peroxidation) through their interaction with KEAP1 [195]. In any case, the most acknowledged cytoplasmic protein able to regulate NRF2 by promoting its stability and nuclear role is p62 [196]. It is a stress-inducible protein involved in multiple signalling pathways, such as autophagy, where it acts as a cargo receptor for the degradation of ubiquitinated proteins and mitochondria, thus preserving organisms from proteotoxic/oxidative stresses [196, 197]. p62 competes with NRF2 for binding to KEAP1, and this interaction induces a dependent autophagic degradation of KEAP1 and subsequent NRF2 stabilization and activation in both MEFs and HEK293 cells [198]. Interestingly, as for BRCA1, p62 expression is regulated by NRF2, due to the presence of ARE sequences in the *p62* promoter, implying a positive-feedback loop [199].

NRF2 activity is also regulated by the availability of many binding partners, including a group of bZIP bi-directional transcription regulators named sMaf proteins (MafF, G and K) [200, 201] which, when bound to NRF2, under OS, favor its maintenance inside the nucleus thereby enhancing its activity [202].

In addition, at the post-translational level, many kinases and acetyl groups are critical for NRF2 subcellular localization and transcriptional activation. Among them, protein kinase C (PKC), a family of protein kinase enzymes involved in various cellular mechanisms, has been shown to phosphorylate NRF2 at Ser40 in its Neh2 domain, disrupting the association with KEAP1 and promoting its activation [203, 204]. Also, the casein kinase 2 (CK2), a highly conserved serine/threonine protein kinase with dual functionality in cellular growth/proliferation and suppression of apoptosis [205], can phosphorylate NRF2 in more than ten sites within Neh4 and Neh5 domains [206], leading to NRF2 translocation to the nucleus and its activation [207]. Alongside these, several studies focused on elucidating the role of mitogen-activated protein kinases (MAPKs) in NRF2 activation, suggested the involvement of c-jun N-terminal kinase 1/2 (JNK1/2) and extracellular signal-regulated kinase 1/2 (ERK1/2) in the NRF2 activation and nuclear accumulation [208, 209]. However, in a later study, it has been demonstrated that direct phosphorylation of NRF2 by MAPKs has a limited contribution in modulating NRF2 activity; rather, MAPKs appear to regulate the NRF2 signalling pathway mainly through indirect mechanisms [210]. Most recently, adenosine monophosphate (AMP)-activated protein kinase (AMPK), a Ser/Thr kinase involved in the cellular response to stressful stimuli [211], has been identified as a novel player in NRF2 activation through its phosphorylation [212]. AMPK phosphorylates NRF2 at the Ser550 residue located in the canonical NES sequence, facilitating NRF2 nuclear accumulation, probably by inhibiting nuclear export [212], and this effect is enhanced by GSK-3β inhibition [213]. Since it is well known that both ubiquitination and phosphorylation are involved in NRF2 regulation, it was no surprise that the acetylation of NRF2 also enhances its activity [214]. It has indeed been demonstrated that p300/CBP transcriptional co-activator proteins (two central players in coordinating and integrating multiple signal dependent events with the transcription apparatus [215, 216]) bind to NRF2 in response to OS induced by arsenite and acetylate several Lys residues within the Neh1, the DNA binding region of NRF2, stabilizing it, increasing protein abundance, and promoting NRF2 nuclear localization [146, 214, 217] (Fig. 5, upper panels).

#### Inhibitors

Pieces of evidence for the transcriptional regulation of NRF2 highlighted that modifications of the *NFE2L2* promoter region such as hypermethylation or single nucleotide polymorphisms, are responsible for a decreased NRF2 expression; however, these findings need further investigation to be confirmed [218–220].

As already discussed above, at the post-transcriptional level, an important role in the regulation of the KEAP1-NRF2 system is played by miRNAs. Many studies revealed the existence of several miRNAs able to target *NFE2L2*, resulting in *NFE2L2* mRNA degradation and inhibition of NRF2 protein translation, such as miR-144, miR-28, miR-93, miR-153, miR-27a, miR142-5p, and miR-34. miR-144 was the first miRNA identified as a NRF2 negative regulator in reticulocytes of patients with homozygous sickle cell disease (HbSS) [221]. Increased levels of miR-144 have been found in a subset of HbSS patients with typical severe anemia and reduced NRF2 protein levels, causing a reduction of GSH regeneration and impairing cell response to OS, thereby providing a possible mechanism for the increased anemia severity seen in these patients [221]. Soon after, a similar inverse association has been found between NRF2 and miR-28 in human MCF7 breast cancer cells, where ectopic expression of miR-28 alone was able to reduce *NFE2L2* mRNA and protein levels, and, as an abnormal miR-28 expression has been seen in a variety of cancers, a role in this regard has been hypothesized [222]. miR-93 is another miRNA able to decrease *NFE2L2* mRNA and protein levels, whose high expression has been associated with human breast carcinogenesis [223]. Subsequently, Mahimainathan’s group identified and validated for the first time a series of miRNAs, miR-153, miR27a, miR142-5p, and also miR-144 in neuronal cells, specifically in human SH-SY5Y neuroblastoma cells, which directly mediate repression of *NFE2L2* in a KEAP1-independent manner [224]. The inhibitory effect on NRF2 activation by miR142-5p was also confirmed by Lv and colleagues using a cellular model of oxygen-glucose deprivation and reoxygenation-induced injury in hippocampal neurons in vitro [225]. Another miRNA shown to regulate Nrf2 is miR-34, able not only to target *NFE2L2* but also downstream genes involved in cellular response to OS, such as *microsomal glutathione S-transferase 1* (*Mgst1*) and *Sirtuin 1* (*Sirt1*) genes in rat liver during aging [226].

In addition to KEAP1, which is the main negative regulator of NRF2 protein stability, many other factors which negatively regulate NRF2 have been discovered and characterized. One of them is GSK-3β, a Ser/Thr protein kinase involved in several metabolic processes, including glycogen metabolism and insulin signalling [213]. Cuadrado and colleagues first showed that GSK-3β inhibits NRF2 activity, but the identification of sites in NRF2 that are phosphorylated by GSK-3β was achieved several years later using two-dimensional electrophoresis and mass spectrometry: GSK-3β phosphorylates a group of Ser residues at the level of DSGIS motif in the Neh6 domain of NRF2, and this event represents the signal for the recognition by the adapter protein β-TrCP, that targets NRF2 for ubiquitination and proteasomal degradation in a KEAP1-independent manner [150, 151]. As discovered more recently by Zhang’s group, NRF2 can also be negatively regulated through the E3 ubiquitin-protein ligase synoviolin (HRD1), recently associated with the ER stress produced by misfolded protein accumulation. HRD1 interacts with the Neh4 and Neh5 domains of NRF2 and causes NRF2 ubiquitination and subsequent degradation through a KEAP1-independent mechanism, just like GSK-3β [227, 228]. Other negative modulators of NRF2 are: CR6-interacting Factor 1 (CRIF1), a protein previously known as a cell cycle regulator and transcription cofactor, which is able to negatively regulate NRF2 protein stability under both reducing and OS conditions through physical interaction with both N- and C-terminal regions of NRF2 [229]; seven in absentia homolog 2 (SIAH2), a key regulator, together with hypoxia-inducible factor 1 alpha (HIF-1α), of the cellular hypoxic responses under pathological conditions such as ischemia-reperfusion, whose association with NRF2 causes the degradation of NRF2 irrespective of its phosphorylation status [230]; and caveolin-1 (CAV1), a scaffold protein of caveolar membranes involved in signal transduction and in the uptake of lipophilic compounds, which directly interacts with NRF2, probably competing with KEAP1 for binding to NRF2, and subsequently suppresses its transcriptional activity [231].

Among the numerous partners that interact with NRF2 regulating its activity, two members of the CNC-bZIP protein family – BTB and CNC homolog 1 (BACH1) and BTB and CNC homolog 2 (BACH2) – are two negative regulators of NRF2: they compete with NRF2 for the binding to ARE sequences of target genes, thereby preventing the production of antioxidant enzymes and cellular defense against toxicity [232, 233].

At the post-translational level, just as CBP/p300-mediated acetylation of Lys within the Neh1 domain increasing NRF2-dependent transcription, SIRT1 mediates Lys deacetylation, antagonizing the NRF2 signal [234]. Interestingly, unlike other MAPKs, such as ERK1/2 and JNK1/2, it has been shown that p38 phosphorylates NRF2 and promotes its association with KEAP1, thereby preventing its nuclear translocation; this effect can be reversed by sulforaphane, a molecule with chemopreventive ability, and, in fact, represents a potential mechanism of action for sulforaphane-mediated induction of NRF2 [235]. Finally, the phosphorylation of tyrosine (Tyr) residues in NRF2 by another kinase, Fyn, present in the nucleus negatively regulates NRF2. More specifically, Fyn phosphorylates NRF2 at Tyr568 leading to nuclear export, ubiquitination, and degradation of NRF2 [236]. It has been demonstrated that GSK-3β acts upstream of Fyn, activating its phosphorylation and nuclear accumulation; in the nucleus Fyn itself can phosphorylate NRF2 and act as a negative regulator [237] (Fig. 5, bottom panels).

### Alteration of KEAP1-NRF2 activity in ALS

The human brain is an organ with elevated demand of energy, relying on oxidative metabolism and high consumption of O_2_ to perform its functions [68]. In addition, non-neuronal cells residing in both the central nervous system (CNS) and peripheral nervous system (PNS) – called glial cells, and among them mainly astrocytes, microglia, and oligodendrocytes – provide essential metabolic and functional support to neurons, contributing to their plasticity [238–241]. Due to their high and variable metabolic and mitochondrial activity, both neurons and astrocytes are especially vulnerable to OS [242].

Therefore, it is essential to activate neuroprotective systems such as NRF2 and its downstream antioxidant signalling, able to counteract pathological insults from highly reactive oxidant species in the brain and limit the accumulation of oxidative damage [68].

Even if it is still unclear whether OS is a primary or a secondary cause of neurodegeneration in ALS, overwhelming evidence indicates the pathological role of OS, and alterations in KEAP1 and NRF2 expression and in KEAP1-NRF2 activity may explain the progressive MN degeneration and death associated with ALS [170]. Dysregulation of KEAP1-NRF2 activity has been observed in ALS cellular and animal models, and confirmed in human ALS tissue [67, 141, 243]. More specifically, NRF2 and KEAP1 have been analyzed in postmortem motor cortex and spinal cord specimens from ALS patients and a reduction in both *NFE2L2* mRNA and NRF2 protein has been found in ALS patient tissues when compared to control ones. The level of *KEAP1* mRNA was shown to be increased in the motor cortex but not in the spinal cord of ALS patients compared to controls; although no significant differences were seen at the protein level [244]. KEAP1 protein has also been shown to co-localise with intracellular inclusions in MNs of postmortem ALS spinal cord [245], possibly through an interaction with p62 observed in several ALS inclusions [246]. The RNA binding protein 45 (RBM45) modulates the antioxidant response in ALS by interacting with KEAP1. Under OS RBM45 nuclear exit occurs (along with cytoplasmic inclusion formation). Once in the cytoplasm, it binds and stabilizes KEAP1, thus interfering with NRF2 stabilization. This in turn results in less NRF2 nuclear entry in response to OS and reduces the cellular antioxidant response, thus contributing to the pathobiology of ALS. Experimental evidence revealed a significant increase in KEAP1-RBM45 binding in cytosolic fractions from ALS lumbar postmortem spinal cord compared to controls; in addition, it has been demonstrated that RBM45 can bind p62 and that RBM45-p62 cytoplasmic colocalization was increased in ALS spinal cords [247]. Nevertheless, as mentioned above, p62 also regulates cell survival via the NRF2 antioxidant response pathway: in particular, the interaction between p62 and KEAP1 is essential for p62-dependent NRF2 signalling [199, 248, 249]. Thus, since several mutations affecting the functional domains of p62 have been identified in patients with ALS and frontotemporal dementia, it has been demonstrated that p62 variants exhibit reduced KEAP1-binding, preventing NRF2 from entering the nucleus and promoting protective genes, and predisposing the cell death upon exposure to ROS [250, 251].

In an effort to clarify the role of the KEAP1-NRF2 pathway in ALS, it has been revealed that the transfection of the mouse MN-like hybrid cell line (NSC-34) with the human SOD1^G93A^ gene causes damage in the Nrf2/ARE signalling and a reduced ability of cells to react to OS. More specifically, cell soma became round, neurites were shorter, and decreased in number, and OS resulted increased. In addition, they found that both the transcript and the protein levels of Nrf2 and the detoxifying/antioxidant enzymes HO1 and NQO1 were significantly decreased [252]. Previously, a study based on proteomic analysis had revealed marked transcriptional repression of other Nrf2-induced antioxidant proteins including CK2, ERK1/2, PKC, GPx, Mgst1, peroxiredoxin 3 (Prdx3), in both preclinical models and in human spinal MNs from ALS patients [253]. These data suggested that pharmacological stimulation of the NRF2/ARE signalling could be a valuable lever to a new therapeutic approach in ALS [253, 254]. More recently, it has been demonstrated that NSC-34 cells expressing TDP-43 mutants also exhibit shortened neurites, increased OS, and decreased HO1 level, and these effects can be reversed by the UPS inhibitor MG132, but not by the Nrf2 activator sulforaphane [255, 256], probably because MG132 induction of HO1 is Nrf2 independent; however, how mutant TDP-43 reduces HO1 level and prevents sulforaphane from activating Nrf2 signalling remains unclear. Similarly to cell lines, primary embryonic MN cultures from SOD1^G93A^ ALS mice showed a diminished Nrf2 nuclear expression and downregulation of the enzymes involved in GSH biosynthesis, associated with increased susceptibility to Nerve Growth Factor (NGF)-induced apoptosis [257]. Surprisingly, extensive down-regulation of miRNAs such as miR-27a, miR-34a, miR-142-5p, and miR-7, involved in cytoprotection against OS and some of them also capable of targeting Nrf2 directly, has been detected in the muscle, CSF, MN progenitors, and blood as well as in post mortem specimens (brain, brain stem, and the spinal cord) of both sALS and fALS patients, unlike healthy controls [258–262]. Such reduction is probably due to a dysregulated miRNA biogenesis under cellular stress and represents a common molecular denominator for multiple forms of human ALS [263].

In contrast to human tissue and cultured cells, it has been demonstrated that Nrf2 activity is consistently elevated in the spinal cord of SOD1^G93A^ rodent models of ALS, and the increase of Nrf2, thioredoxin, HSP-70, HO1, NQO1, GCLC, and GCLM protein levels follows disease progression in the lumbar spinal cord but not cortex [264, 265]. Interestingly, both Nrf2 and HO1 co-localized with reactive astrocytes, the major GSH suppliers for neighboring neurons, in the degenerating spinal cord of SOD1^G93A^ rats, and this was interpreted as a reactive attempt to prevent cell death; moreover, crossing SOD1^G93A^ mice with mice overexpressing Nrf2 selectively in astrocytes significantly delayed disease onset and extended survival, thus reversing the toxicity of astrocytes expressing human SOD1^G93A^ mutation to co-cultured MNs [266, 267]. Furthermore, it has been found that modulation of the Nrf2 signalling pathway by triterpenoids compounds results in a significant increase in survival in the SOD1^G93A^ mouse model of ALS, thereby indicating that activation of Nrf2 has a neuroprotective effect [268]. In addition, cross-breeding ALS-transgenic mice with ARE-human placental alkaline phosphatase (hPAP) reporter mice allowed to reveal that Nrf2 activity was more intense in skeletal muscle than in the spinal cord, and evident since before motor symptom onset [264]. These data were in line with the hypothesis that neuromuscular junctions, which are the synapses between MNs and muscle fiber, could represent the starting site of MN dysfunction in ALS [269]. However, contrary to expectations, the deletion of the *Nfe2l2* gene had a moderate impact on the course of the disease in SOD1^G93A^ mice and negatively affected only a few Nrf2-regulated antioxidant enzymes, thus suggesting that several Nrf2-target genes can also be regulated independently of Nrf2 in ALS mice [270, 271].

### KEAP1-NRF2 system as a potential therapeutic target in ALS

The presence in ALS of increased levels of OS markers and impaired antioxidant defense in the brain and peripheral tissues [25] together with the central role of NRF2 in inducing target genes to counteract OS [272] make the KEAP1-NRF2 system a suitable therapeutic target for drugs and small molecules.

Various chemical inducers of the NRF2 activity have been identified in the last two decades, but several of these compounds still have significant drawbacks associated to their clinical potential: suboptimal pharmacokinetics (concentration vs. time) and pharmacodynamics (effect vs. time), poor target selectivity, different efficacy between animal models and human pathologies, and safety issues [66].

The main NRF2-activating drug candidates that have been explored, and many of which have reached different stages of clinical trials, are presented in Table 1.

**Table 1:** Summary of the drug development status of NRF2 activators

Compound	Mechanism of action	Disease	Development stage	Trial/References
KEAP1-dependent NRF2 activators				
Electrophilic compounds				
Cyanoenone triterpenoids.				
Bardoxolone methyl (BARD, CDDO-Me, RTA402)	Modification of C151 in KEAP1	Alport syndrome	Phase II/III (active)	CARDINAL/NCT03019185
		Autosomal dominant polycystic kidney disease	Phase II (completed)	PHOENIX/NCT03366337
		PAH	Phase III (active)	RANGER/NCT03068130
Omaveloxolone	Modification of C151 in KEAP1	Diabetic chronic non-healing wounds	Preclinical	[277]
		FRDA	Phase II (active)	MOXIe/NCT02255435 https://clinicaltrials.gov/ct2/show/NCT02255435
Fumaric acid esters.				
Dimethylfumarate (DMF)	Modification of C151 in KEAP1	Psoriasis	Approved	[281]
		MS	Approved	PROTEC/NCT01930708
				https://www.ema.europa.eu/en/medicines/human/EPAR/tecfidera
VCB102	Modification of C151 in KEAP1	Psoriasis	Preclinical	V ClinBio LLC
VCB101	Modification of C151 in KEAP1	MS	Preclinical	V ClinBio LLC
CAT4001	Modification of C151 in KEAP1	FRDA	Preclinical	Catabasis Pharmaceuticals
XP23829	Modification of C151 in KEAP1	Psoriasis	Phase II (completed)	NCT02173301
ALK8700/BIIB098	Modification of C151 in KEAP1	MS	Phase III (completed)	EVOLVE-MS-2/NCT03093324
Hydroxylamine.				
N-tert-butyl hydroxylamine	Targeting of KEAP1	Retinal pigment epithelial cells	in vitro	[285]
OT551	Targeting of KEAP1	Age-related macular degeneration	Phase II (completed)	OMEGA/NCT00485394
Nitro fatty acids.				
CXA10	Modification of C273 and C288 in KEAP1	PAH	Phase II (completed)	PRIMEx/NCT03449524
		Primary focal segmental glomerulosclerosis	Phase II (completed)	FIRSTx/NCT03422510
NATOH, NATxME and NATx0	Modification of C273 and C288 in KEAP1	Inflammation related diseases	in vitro/Preclinical	[288]
Sulforaphane.				
Sulforaphane (SFN)	Modification of C151 in KEAP1	Autism spectrum disorder	Phase II (active)	NCT02677051
		Hypoxic-ischemic injury, AD, PD	Preclinical	[292]
Melatonin–sulforaphane hybrid (ITH12674)	Modification of C151 in KEAP1	Neuronal OS	in vitro	[293]
SFX-01	Modification of C151 in KEAP1	ER^+^ metastatic breast cancer	Phase II (completed)	STEM/NCT02970682
		Subarachnoid hemorrhage	Phase II (completed)	SAS/NCT02614742
TFM735.				
TFM735	Modification of C151 in KEAP1	EAE models of MS	Preclinical	[297]
Non-electrophilic compounds				
Naphthalene bis-sulfonamides, Tetrahydroisoquinolines, and Molecules with an oxa-diazole motif	KEAP1-NRF2 PPI inhibition	COPD	in vitro/Preclinical	[298]
DEETGE-CAL-Tat synthetic peptide	KEAP1-NRF2 PPI inhibition	Brain injury	Preclinical	[301]
		GCI	Preclinical	[302]
KEAP1-independent NRF2 activators				
BACH1 inhibitors.				
HPP-4382	BACH1 inhibition	Lung fibroblasts	in vitro	[303]
HPP971	BACH1 inhibition	EAE models of MS	Phase I (completed)	[304]
		Blood, Bone, Eye, Kidney, and Lung diseases	Phase II (completed)	vTv Therapeutics https://vtvtherapeutics.com/pipeline/nrf2-bach1-program/
HRD1 inhibitors.				
LS-102	HRD1 inhibition	Liver cirrhosis	in vitro/Preclinical	[228]
GSK-3β inhibitors.				
Nordihydroguaiaretic acid	GSK3-β inhibition	Prostate cancer	Phase II (completed)	NCT00678015
		Brain and CNS tumors	Phase I/II (completed)	NCT00404248
Terameprocol	GSK3-β inhibition	High grade glioma	Phase I (active)	NCT02575794
Enzastaurin	GSK3-β inhibition	Diffuse large B-Cell lymphoma	Phase III (active)	NCT03263026
p62 activators.				
Trehalose	p62 activation	Hepatoma cells	in vitro	[308]
Rapamycin	p62 activation	FRDA	Preclinical	[309]
		Systemic lupus erythematosus	Phase II (completed)	NCT00779194
		Diabetes mellitus type1	Phase III (completed)	NCT01060605
		Autosomal dominant polycystic kidney disease	Phase II (stopped)	NCT00920309
		ALS	Phase II (active)	RAP-ALS/NCT03359538

### KEAP1-dependent NRF2 activators

#### Electrophilic compounds

The most known NRF2 activators are electrophiles – electron-deficient species able to form covalent bonds with electron-rich nucleophiles through a variety of chemical pathways – which can covalently bind and modify cysteine residues in KEAP1 [273, 274].

#### Cyanoenone triterpenoids

This class of synthetic pentacyclic triterpenoids derives from the natural compound oleanolic acid [268, 275]**,** primarily reacts with C151 in KEAP1 by interrupting KEAP1-CUL3 interaction [123], and includes the strongest NRF2 activators currently known [276]. One of them, bardoxolone methyl (BARD, CDDO-Me, RTA402) is in the late stages of clinical trials for several conditions related to advanced chronic kidney disease – including Alport syndrome and an autosomal dominant polycystic kidney disease – and to pulmonary arterial hypertension (PAH). The efficacy of a second-generation derivative, omaveloxolone (RTA408), has recently been assessed in a preclinical model for diabetic chronic non-healing wounds [277] and, interestingly, omaveloxolone is now being tested in phase-II clinical trial for the Friedreich’s ataxia (FRDA), a neurodegenerative condition in which NRF2 activation is suppressed [278, 279].

#### Fumaric acid esters

Fumaric acid esters are the most investigated NRF2 activators. These electrophilic modulators mainly interact with C151 in KEAP1, thereby inhibiting NRF2 ubiquitination and increasing the activity of ARE-mediated antioxidant and detoxifying enzymes [280]. Among them, the most clinically successful NRF2 activator is dimethylfumarate (DMF), authorized in Germany in 1994 for the treatment of psoriasis [281]. It was clinically approved by both the Food and Drug Administration (FDA) and the European Medicine Agency (EMA) in 2014 for the treatment of relapsing-remitting multiple sclerosis (MS), showing a significant efficacy over 2 years versus placebo and a favorable benefit-risk profile in two phase-III clinical trials, although with several adverse effects such as nausea, diarrhea, abdominal pain and leukopenia in some patients [282, 283]. Since DMF is metabolized by intestinal esterases to monomethylfumarate (MMF), novel MMF derivative compounds with greater efficacy, bioavailability, and reduced side effects are currently under investigation in preclinical studies, including VCB102 compound for psoriasis, and VCB101 compound for MS. In addition, CAT4001 compound has been shown to decrease ROS production, normalize mitochondrial length, and improve mitochondrial function in dorsal root ganglion (DRG) derived neurons from FRDA’s mice [284], thus expanding the therapeutic potential also in the field of neurodegenerative diseases. Furthermore, two other MMF derivatives are being tested in phase-II or -III clinical trials for psoriasis – XP23829 compound, and for MS – ALK8700/BIIB098 compound.

#### Hydroxylamine

Hydroxylamine is a synthetic NRF2 activator that protects against OS by targeting KEAP1. Several substituted derivatives of hydroxylamine are known and, it has been shown that one of them, N-tert-butyl hydroxylamine, protects cells from OS and mitochondrial damage in an in vitro model of age-related macular degeneration (AMD) [285]. In addition, most recently, another di-substituted hydroxylamine, named OT551, able to cross blood-brain barrier (BBB) and inhibit OS and inflammation, has been developed and has shown efficacy in a phase-II clinical trial on age-related macular degeneration.

#### Nitro fatty acids

Nitro-fatty acids (NO_2_-FAs) are endogenous signalling mediators with strong anti-fibrotic and anti-inflammatory activities [286], able to promote NRF2 activation through the interaction and reversible post-translational modification of C273 and C288 present in the stress sensing domain (IVR) of KEAP1 [287]. Two phase-II clinical trials have just been completed to test the efficacy of CXA10 compound as a potential treatment for PAH and primary focal segmental glomerulosclerosis. Moreover, novel nitroalkenes derived from α-tocopherol (NATOH, NATxME, and NATx0) are being characterized in both in vitro and in vivo models [288], thus opening the way to new therapeutic strategies.

#### Sulforaphane

Several natural electrophilic activating compounds of NRF2 have been identified, including epigallocatechin 3-gallate, quercetin, α-lipoic acid, and sulforaphane (SFN) [289]. The last one is an organo-sulfur compound first identified in broccoli [290] able to modify C151 in KEAP1, therefore inducing the antioxidant NRF2-target enzyme NQO1 [158]. A phase-II clinical study currently underway will evaluate if sulforaphane improves core symptoms in patients with autism spectrum disorders [291]. A very important aspect of this compound is that it can cross BBB and it is able to protect cells from OS in many preclinical models of neurologic diseases, such as hypoxic-ischemic injury, AD, PD [292]. In addition, in an attempt to have a dual ‘drug-prodrug’ mechanism of action, a melatonin-SFN hybrid has been designed, which demonstrated to induce neuroprotection in cortical neurons subjected to OS [293]. Furthermore, to improve the stability of SFN, a cyclodextrin formulation, SFX-01, has been developed for which two phase-II clinical trials have just been completed, evaluating the safety and efficacy in the field of ER^+^ metastatic breast cancer and subarachnoid hemorrhage [294, 295].

#### TFM735

This compound was identified by high-throughput screening analysis as an activator of NRF2 through a C151-dependent mechanism [296]. TFM735 was shown to inhibit T cell proliferation and improve experimental autoimmune encephalomyelitis (EAE) in a mouse model of MS [297].

### Non-electrophilic compounds

Also known as KEAP1-NRF2 protein-protein interaction (PPI) inhibitors, they comprise several classes of compounds, including naphthalene bis-sulfonamides, tetrahydroisoquinolines, and molecules that present an oxadiazole motif [66]. Evidence has shown their capability to increase the expression of NRF2 target genes in cellular and in vivo models of chronic obstructive pulmonary disease (COPD) [298], and to interfere with the direct PPI between KEAP1 and NRF2 or the PPI between KEAP1 and CUL3 [299, 300]. It had previously been shown that the amino acid sequence DEETGE is essential for NRF2-KEAP1 interaction [124]. To increase cell penetrance, the DEETGE motif was fused with the Tat sequence of the human immunodeficiency virus together with the cleavage sequence of calpain (Cal). The intracerebroventricular (ICV) injection of the DEETGE-Cal-Tat peptide showed an increased expression of Nrf2- regulated genes and marked neuroprotective and cognitive-preserving effects in mice subjected to brain injury [301]. Similarly, ICV pretreatment or peripheral post-treatment with the DEETGE-Cal-Tat peptide was also able to decrease Keap1-Nrf2 interaction in the rat hippocampal CA1 region after global cerebral ischemia (GCI) and, as a consequence, to induce Nrf2 target genes and a powerful neuroprotection of hippocampal-dependent cognitive function after GCI [302].

Although several PPI inhibitors with improved selectivity have recently been identified, using combined approaches that brought together protein crystallography, ligand NMR spectroscopy, and computational chemistry, unfortunately, none of them has been found to be able to cross the BBB and many are quite large in size and contain carboxylic acids, features which usually prevent or decrease CNS permeability [299].

### KEAP1-independent NRF2 activators

Given the complex regulation of the KEAP1-NRF2 system, it is not surprising that new pharmacological strategies are being explored, aimed at inducing NRF2 activity also in a KEAP1-independent manner.

#### BACH1 inhibitors

One member (HPP-4382) of the synthetic small molecules targeting BACH1 was able to induce the antioxidant enzyme HO1 through NRF2 activation in human lung fibroblasts [303]. Another compound, HPP971, elevated reduced GSH levels and protected human astrocytes from H_2_O_2_-induced cell death in vitro and induced HO1 expression in a murine EAE model of MS, attenuating loss of motor functions [304]. Furthermore, HPP971 has completed two phase-I studies for the treatment of several diseases, including blood, bone, eye, kidney, and lung diseases, where it was well tolerated [66].

#### HRD1 inhibitors

Pharmacological inhibition of HRD1, a negative regulator of NRF2 implicated in its ubiquitination and degradation, was tested in human liver tissues and in an animal model for the liver cirrhosis characterized by increased ER stress and ROS: LS-102 alleviated liver cirrhosis enhancing NRF2 signalling pathway [228].

#### GSK-3β inhibitors

GSK-3β regulates multiple critical intracellular signalling pathways and it has been implicated in the pathogenesis of numerous diseases, including cancer [305], myocardial diseases [306], and neurodegenerative diseases [307]. In the last years, several preclinical studies and clinical trials have been effectuated using different GSK-3β inhibitors, actually with few encouraging results. Among these compounds, nordihydroguaiaretic acid showed no significant effects in two phase-II clinical trials for the treatment of prostate cancer and CNS tumors. Most recently, terameprocol reached phase I clinical trial for the treatment of high-grade glioma, still ongoing. In addition, a new phase-III clinical trial was started two years ago to evaluate the effects of enzastaurin in patients with diffuse large B-cell lymphoma.

#### p62 activators

Two compounds able to increase p62 levels, trehalose and rapamycin, have been demonstrated to increase nuclear translocation of NRF2 in a p62-dependent manner and to enhance expression of its downstream antioxidant factors, HO1 and NQO1, in a hepatoma cellular system [308] and in a preclinical model of FRDA [309]. These compounds have been also tested in two now completed phase-II and -III trials for the treatment of systemic lupus erythematosus [310] and diabetes mellitus type 1 [311] respectively, showing progressive improvement in disease activity thereby opening the way for new treatment perspectives. Unfortunately, another phase-II/III clinical study for the treatment of autosomal dominant polycystic kidney disease was stopped because it failed to show clinical benefits to patients. A multi-center phase-II clinical trial, started a few years ago to investigate the biological and clinical effects of rapamycin in people with ALS, is currently underway and will provide important information for further potential trials [312], thus opening new therapeutic perspectives for ALS.

## Conclusions

High levels of OS can be considered a result of the imbalance between oxidative species and antioxidant defense systems, and indeed represent a hallmark of many acute and chronic diseases, including ALS. In this context, it is now widely recognized that the KEAP1-NRF2 system, which is essential for the maintenance of redox homeostasis, is impaired in ALS. The structural organization and functionality of these two players, under physiological conditions and in response to OS, present a tight regulation at multiple levels and involve different partners. Evidence has determined that elevated oxidative damage to proteins, lipids, and nucleic acids is a distinctive characteristic of the motor cortex and spinal cord of ALS patients, together with high levels of KEAP1 and a decrease in the NRF2-target genes and related enzymes.

Dysregulation of KEAP1-NRF2 activity has been extensively demonstrated in cellular and animal models of ALS and in postmortem ALS motor cortex and spinal cord samples, so dysregulation of the NRF2/ARE antioxidant and cytoprotective pathway could be a possible mechanism underlying the progressive neurodegeneration in ALS and severity of the disease. Since NRF2 activation induces the antioxidant and detoxifying response to restore redox homeostasis state, in recent years there has been a growing interest in identifying and evaluating new compounds capable of targeting the KEAP1-NRF2 system, to induce cytoprotective response against OS. A variety of NRF2 activators, both KEAP1-dependent and -independent compounds, have been identified and several of them are currently in clinical development. Some of these compounds have promising therapeutic effects in animal models and in several clinical trials for various pathologies including chronic neurological diseases. Nonetheless, to date the only FDA- and EMA-approved NRF2 activator for the treatment of relapsing-remitting MS and psoriasis is DMF, and its therapeutic potential in ALS patients in terms of efficacy, safety and tolerability, is now being evaluated in a phase-II clinical trial [313].

Despite many advances in the attempt to activate NRF2 through different pharmacological approaches, much remains to be done to fully benefit from the various drug candidates able to modulate the cellular antioxidant response mediated by NRF2. In particular, the suboptimal pharmacokinetics and pharmacodynamics of these compounds and their poor ability to cross the BBB could represent considerable obstacles for a significant therapeutic efficacy. In the last decade, studies on the condition of BBB in neurodegenerative diseases, including ALS, have demonstrated the presence of vascular disruption, a condition that could theoretically favour the entry of drugs into brain regions affected by neurodegeneration. However, it has been found that the pathological degradation of BBB hinders the proper delivery and action of therapeutic agents, due to a series of functional, structural, and biochemical changes at both the endothelial level and, consequently, in the perivascular and interstitial space [314]. This background suggests that the optimal condition for a drug to be more efficient in carrying out its therapeutic action would be to cross a BBB with good vascular integrity and without further damage in the neuronal functional state. This would be especially true for drugs relying on a fine functional remodulation of a well preserved cellular system, like the NRF2 activators on the KEAP1-NRF2 system. Thus, studies on the combination of strategies that can penetrate a normal BBB or on the dynamics of BBB disruption, as well as on the identification of biomarkers of brain vascular injury, might pave the way to new drug delivery approaches targeting BBB in the near future.

**Fig. 6:**
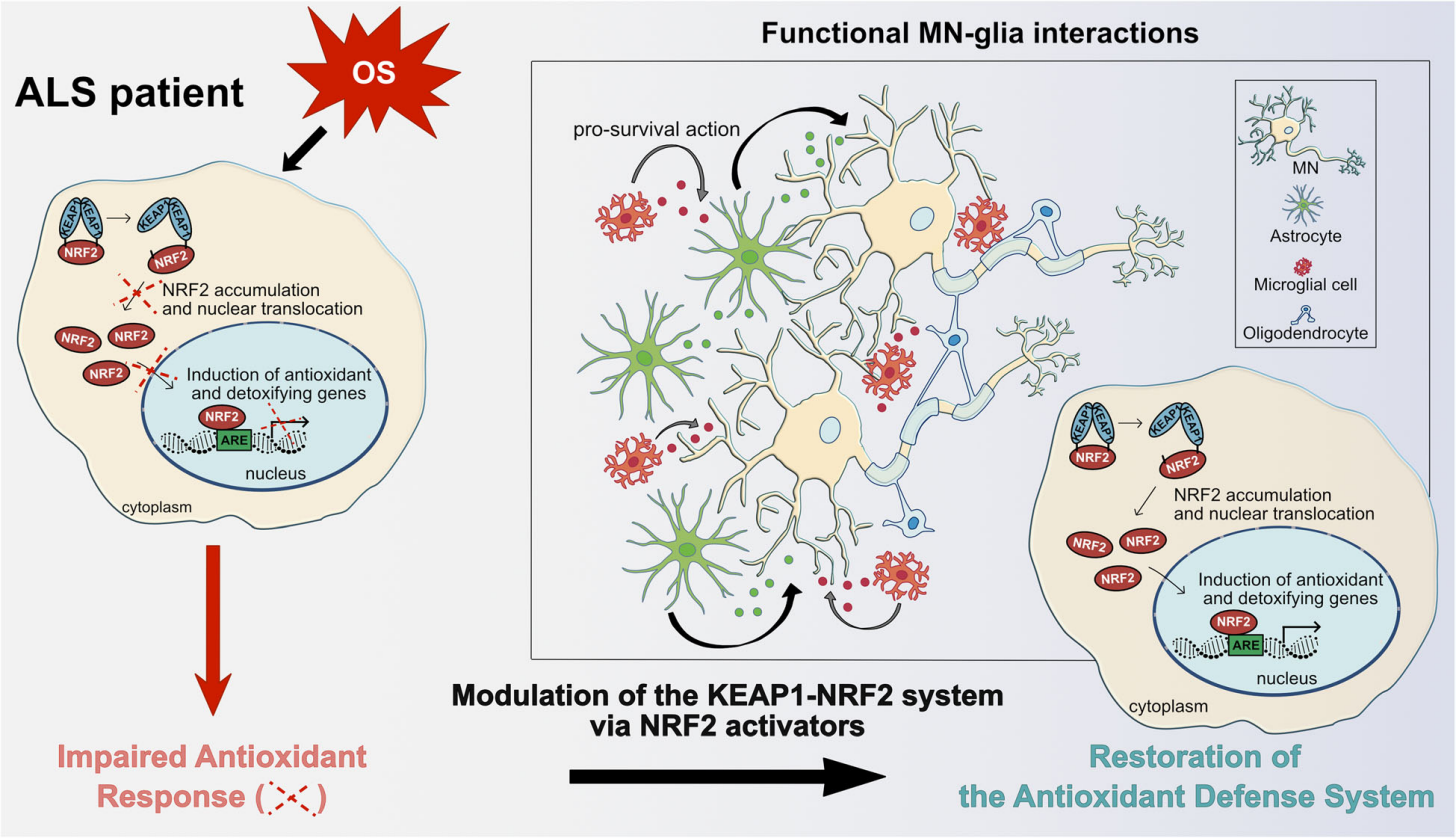
Activation of the NRF2 signalling as a potential treatment for ALS

Furthermore, as specified above, MNs are not isolated units and also rely on glial cells for antioxidant protection through the NRF2/ARE signalling; in fact, recent evidence has shown that boosting astrocytic Nrf2 can have protective effects both in ALS mouse models and in vitro cultures. The fact that NRF2 is readily inducible in cultured astrocytes suggests that most pharmacological NRF2 activators may specifically target astrocytes in vivo, although there is still limited direct evidence for this. In addition, given that microglia clearly exhibit functional NRF2 signalling [315], it could be an important target for NRF2 induction in neurodegeneration. However, there are few data on NRF2 activation in microglia in human tissues or animal models of ALS, and NRF2 signaling has not yet been evaluated in oligodendrocytes, although this may be relevant in the case of ALS which also involves demyelination [316]. Therefore, it would be crucial to understand and outline the specific contribution of each cell type – MN, glia, or others – to ALS, in order to strategically target them with the NRF2 activators, thereby restoring neuronal homeostasis and survival, and functional MN-glia interactions (Fig. 6).

As NRF2 enhances cell survival under stress, we can expect that increased NRF2 activity may promote tumorigenesis through stress protection [317, 318]. Dodson and colleagues have clearly reviewed how complex it is to target NRF2 in diseases [319]. In particular, they pointed out that although NRF2 has been traditionally considered as a tumor suppressor because of its cytoprotective functions [320], a growing number of studies have shown a strong NRF2 activation at certain stages of cancer [318, 321]. Another study suggested the existence of a correlation between NRF2 accumulation and the development of a multisystemic disorder, characterized by immunodeficiency and neurological symptoms [322]. In this light, we want to emphasize how a timely and appropriately regulated manipulation of the KEAP1-NRF2 system is crucial to develop effective and safe therapeutic treatment for neurodegenerative diseases, such as ALS, which involve an imbalance in the OS response. Therefore, while aware that the pharmacological modulation of the NRF2/ARE pathway could undeniably offer new therapeutic opportunities for diseases related to OS, we should at the same time consider that most of the currently known activators of NRF2 are not particularly specific, leading to an increased risk of “off target” toxic effects. It follows that an efficient modulator of the KEAP1-NRF2 system should be set, not only with significant efficacy and bioavailability, but also with high specificity [323].

Recently, population-based studies have revealed that ALS is an age-related disease similar to other age-associated neurodegenerative diseases like PD and AD [324]. Both aging and neurodegenerative disorders, such as ALS, share a multifactorial process underlying their progressive evolution, which is genetically determined and epigenetically influenced by the environment. Nonetheless, the existence of a biological interplay among OS, aging, and ALS has been presumed for more than two decades [325] and it is now becoming more evident [326]. Thus, in the course of aging various potentially pathogenic mechanisms, including the increase in oxidative damage, could on the one hand influence the quality of aging and on the other favour the onset and evolution of a definite neurodegenerative disease. Although with some controversy, the accumulated evidence directly supports an age-related decline in the ability to respond to OS with the activation of the NRF2/ARE signalling pathway, thereby reducing the expression of its target antioxidant genes [327]. However, the phenomena that induce OS attributable to the patient’s aging, in addition to being variable in terms of intensity among individuals, are difficult to distinguish from those produced by the compromised antioxidant defense systems due to a neurodegenerative disease, such as ALS. It follows that decoding the precise relationship between aging and ALS could allow to understand whether the effects of aging, including OS, might be a prerequisite to developing the MN disease [326].

In this regard, it would be interesting to evaluate if and which OS markers may directly or indirectly reflect the inadequate functioning of the KEAP1-NRF2 system in counteracting OS, and where they are already present at high levels in young adults. Indeed, conceptually, it would be critical to monitor the progressive pathological changes of these markers throughout adulthood, so to identify subjects possibly at risk of developing a neurodegenerative process favoured by the presence of OS induced, at least in part, by an imbalance of the KEAP1-NRF2 system. However, no single OS marker is representative in determining how healthy an individual is, or in predicting his life expectancy, while a set of clinical biomarkers, including lipid peroxidation and protein oxidation products, and antioxidative acting enzymes, could be used to delineate the oxidation status of a person [328].

As a matter of fact, taking into full account the intricacy of the numerous regulatory mechanisms involved in OS control as well as the complex interplay between the many events that contribute to the progressive degeneration and death of MNs, future neuroprotective pharmacological therapies will likely need to aim for an integrated treatment, acting on several levels to limit the progression of the disease. An adequate and hopefully incisive therapeutic action will therefore depend on a finely orchestrated balance between the efficacy of the single treatment for the specific pathogenetic mechanism and the control of potentially noxious interactions among the different therapies. In that sense, while still representing a challenging goal to be achieved, the identification of specific prognostic biomarkers and also those for the evaluation of therapeutic efficacy in ALS will have a fundamental role in designing and monitoring new treatment strategies in the complex scenario of the neurodegenerative process.

## Data Availability

Not applicable.

## Abbreviations

3-NT: 3-nitrotyrosine
4-HNE: 4-hydroxy-nonenal
8-OHdG: 8- hydroxy-2′-deoxyguanosine
AhR: aryl hydrocarbon receptor
AKT: protein kinase B
AMP: adenosine monophosphate
AMPK: AMP-activated protein kinase
ARE: antioxidant responsive element
BACH1: broad complex-tramtrack-bric à brac and cap ‘n’ collar homolog 1
BACH2: broad complex-tramtrack-bric à brac and cap ‘n’ collar homolog 2
BBB: blood-brain barrier
BRCA1: breast cancer susceptibility gene 1
BTB: Broad complex/Tramtrack/Bric à brac
bZIP: basic region leucine zipper
C9orf72: chromosome 9 open reading frame 72
Cal: calpain
CAT: catalase
CAV1: caveolin-1
CBP: CREB-binding protein
CHD6: chromo-ATPase/helicase DNA-binding protein 6
CK2: casein kinase 2
CNC: cap ‘n’ collar
CNS: central nervous system
CREB: cAMP response element-binding protein
CRIF1: CR6-interacting Factor 1
CRM1: chromosomal region maintenance 1
CSF: cerebrospinal fluid
CUL3: cullin 3
DEM: diethyl maleate
DGR: Double glycine repeat
DLG: Aspartic acid-Leucine-Glycine
DMF: dimethylfumarate
DPP3: dipeptidyl-peptidase 3
DSAPGS: Aspartic acid-Serine-Alanine-Proline-Glycine-Serine
DSGIS: Aspartic acid-Serine-Glycine-Isoleucine-Serine
ERK1/2: extracellular signal-regulated kinase 1/2
ETGE: Glutamic acid-Threonine-Glycine-Glutamic acid
FUS: fused in Sarcoma
GBE: glycogen branching enzyme
GCL: glutamate-cysteine ligase
GCLM: glutamate-cysteine ligase modifier
GPx: glutathione peroxidase
GSH: glutathione
GSK-3β: glycogen synthase kinase-3 beta
H_2_O_2_: hydrogen peroxide
HIF-1α: hypoxia-inducible factor 1 alpha
HO1: heme oxygenase 1
^•^HO_2_: hydroperoxyl radical
H_2_O_2_: hydrogen peroxide
hPAP: human placental alkaline phosphatase
HRD1: synoviolin
IVR: central intervening region
JNK1/2: c-jun N-terminal kinase 1/2
KEAP1: Kelch-like ECH (erythroid cell-derived protein with CNC homology)-associated protein 1
MAPKs: mitogen-activated protein kinases
MDA: malondialdehyde
MEFs: mouse embryonic fibroblasts
Mgst1: microsomal glutathione S-transferase 1
miRNA: microRNA
MMF: monomethylfumarate
NAPDH: nicotinamide adenine dinucleotide phosphate
NEDD8: neural precursor cell expressed, developmentally down-regulated 8
Neh: Nrf2-ECH homology
NES: Nuclear Export Signal
NLS: Nuclear Localization Signal
NFE2L2: Nuclear factor erythroid 2 like 2 gene
NF-κB: nuclear factor kappa-light-chain-enhancer of activated B cells
NGF: Nerve Growth Factor
NO^•^: nitric oxide radical
^•^NO_2_: nitrogen dioxide radical
NOTCH1: neurogenic locus notch homolog protein 1
NPC: nuclear pore complex
NQO1: NADPH quinone dehydrogenase
NRF2: nuclear factor erythroid 2-related factor 2
O_2_: molecular oxygen
O_2_^•-^: superoxide anion
^•^OH: hydroxyl radical
ONOO^−^: peroxynitrite
OS: oxidative stress
OXPHOS: oxidative phosphorylation
PALB2: partner and localizer of BRCA2
PGAM5: phosphoglycerate mutase 5
PHKA1: phosphorylase b kinase subunit A1
PI3K: phosphoinositide 3-kinase
PKC: protein kinase C
PNS: peripheral nervous system
ProTα: prothymosin alpha
RAC3: receptor-associated coactivator 3
RBM45: RNA binding protein 45
RBX1: RING box protein 1
RNS: reactive nitrogen species
^•^ROO: peroxyl radical
ROS: reactive oxygen species
RS^•^: thiyl radicals
RSOH: sulfenic acid
RS(O) SR: thiosulfinate
RS(O_2_) SR: thiosulfonate
RSS: reactive sulfur species
RSSR: disulfides
RXR*α*: retinoid X receptor alpha
SFN: sulforaphane
SIAH2: seven in absentia homolog 2
Sirt1: Sirtuin 1
sMaf: small musculoaponeurotic fibrosarcoma
SOD1: superoxide dismutase 1
SQSTM1: sequestosome-1 gene
TARDBP: TAR DNA-binding protein 43 gene
TCTP: translationally controlled tumor protein
TDP-43: TAR DNA-binding protein 43
β-TrCP: β-transducin repeat-containing protein
Ub: ubiquitin
UCH-L1: ubiquitin carboxyl-terminal hydrolase isoenzyme L1
UPS: ubiquitin-proteasome system
XRE: xenobiotic response element
